# Relationship between door-to-embolization time and clinical outcomes after transarterial embolization in trauma patients with complex pelvic fracture

**DOI:** 10.1007/s00068-021-01601-7

**Published:** 2021-02-01

**Authors:** Hohyun Kim, Chang Ho Jeon, Jae Hun Kim, Hoon Kwon, Chang Won Kim, Gil Hwan Kim, Chan Kyu Lee, Sang Bong Lee, Jae Hoon Jang, Seon Hee Kim, Chan Yong Park, Seok Ran Yeom

**Affiliations:** 1 Department of Trauma Surgery and Surgical Critical Care, Pusan National University Hospital, 179 Gudeok-Ro, Seo-Gu, Busan, 49241 Korea; 2 Biomedical Research Institute, Pusan National University Hospital, Busan, Korea; 3 Pusan National University School of Medicine, Yangsan-si, Gyeongsangnam-do Korea; 4 Department of Diagnostic Radiology, Pusan National University Hospital, Busan, Korea; 5 Department of Orthopaedic Surgery, Pusan National University Hospital, Busan, Korea; 6 Department of Trauma Surgery, Wonkwang University Hospital, Iksan-si, Jeollabuk-do Korea; 7 Department of Emergency Medicine, Pusan National University Hospital, Busan, Korea

**Keywords:** Multiple trauma, Therapeutic embolization, Pelvic bone, Mortality rate, Time

## Abstract

**Background:**

While transarterial embolization (TAE) is an effective way to control arterial bleeding associated with pelvic fracture, the clinical outcomes according to door-to-embolization (DTE) time are unclear. This study investigated how DTE time affects outcomes in patients with severe pelvic fracture.

**Methods:**

Using a trauma database between November 1, 2015 and December 31, 2019, trauma patients undergoing TAE were retrospectively reviewed. The final study population included 192 patients treated with TAE. The relationships between DTE time and patients’ outcomes were evaluated. Multiple binomial logistic regression analyses, multiple linear regression analyses, and Cox hazard proportional regression analyses were performed to estimate the impacts of DTE time on clinical outcomes.

**Results:**

The median DTE time was 150 min (interquartile range, 121–184). The mortality rates in the first 24 h and overall were 3.7% and 14.6%, respectively. DTE time served as an independent risk factor for mortality in the first 24 h (adjusted odds ratio = 2.00, 95% confidence interval [CI] = 1.20–3.34, *p* = 0.008). In Cox proportional hazards regression analyses, the adjusted hazard ratio of DTE time for mortality at 28 days was 1.24 (95% CI = 1.04–1.47, *p* = 0.014). In addition, there was a positive relationship between DTE time and requirement for packed red blood cell transfusion during the initial 24 h and a negative relationship between DTE time and ICU-free days to day 28.

**Conclusion:**

Shorter DTE time was associated with better survival in the first 24 h, as well as other clinical outcomes, in patients with complex pelvic fracture who underwent TAE. Efforts to minimize DTE time are recommended to improve the clinical outcomes in patients with pelvic fracture treated with TAE.

**Supplementary Information:**

The online version contains supplementary material available at 10.1007/s00068-021-01601-7.

## Background

The incidence of pelvic fracture in blunt trauma is as high as 10%, with mortality ranging from 21 to 50% primarily owing to hemorrhagic shock [1, 2]. Hemorrhage from pelvic vessels is a dreaded and potentially lethal condition of pelvic fractures [3, 4]. Pelvic transarterial embolization (TAE) is the most effective intervention for management of arterial hemorrhage associated with pelvic fracture [5–7]. TAE has come of age and has an important role in the treatment of patients with pelvic fracture, supported by the highest level of evidence [7–9]; for instance, pelvic angiography with embolization seems to be 85–97% effective for controlling bleeding [6, 8].

Delayed hemorrhage control may increase a patient’s mortality risk with time; early angiography with embolization is associated with improved patient outcomes in patients with pelvic fracture [10–13]. However, many previous studies have shown that it is difficult to achieve this goal [14, 15].

The current study evaluated the impact of delays in performing pelvic TAE on patients’ survival. We hypothesized that a larger door-to-embolization (DTE) time would be significantly associated with increased mortality in patients with complex pelvic fracture.

## Methods

### Study setting

**Fig. 1:**
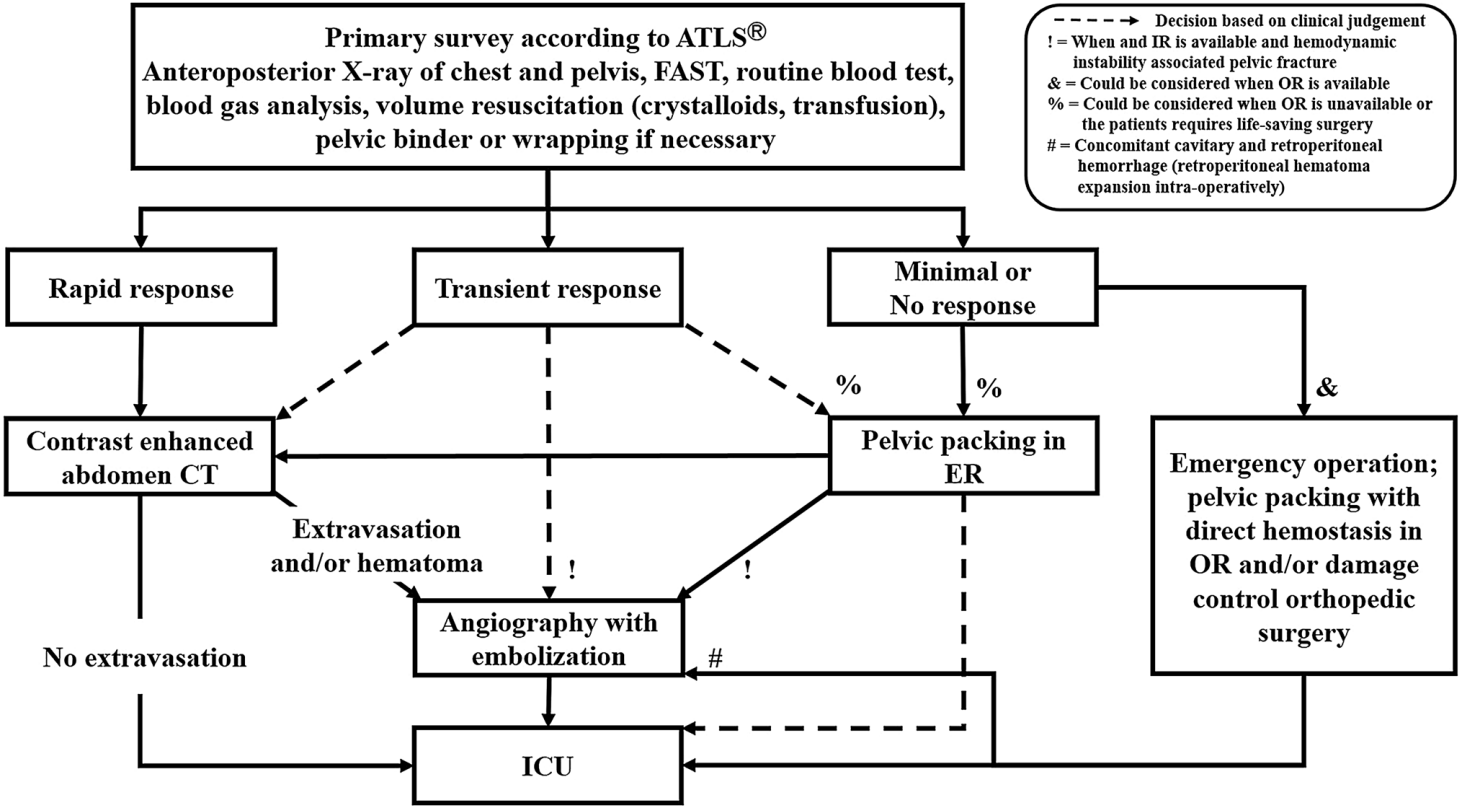
Pelvic fracture management algorithm. *ATLS* adult trauma life support, *FAST* focused assessment with sonography in trauma, *ER* emergency room, *IR* interventional room, *OR* operating room, *ICU* intensive care unit

In the Pusan National University Hospital Regional Trauma Center, there are more than 900–1,000 severe trauma-related admissions annually (Injury Severity Score [ISS] ≥ 16), of which 200–250 patients present with pelvic fracture. Our institution is equipped with a trauma bay, a 42-bed dedicated trauma intensive care unit (ICU), and a trauma angiography suite. Three interventional radiologists and the equipment required for TAE are available 24 h a day, 7 days a week [16]. Thus, the time from arrival to angiography can be less than 2 h. Patients with pelvic fractures without extrapelvic injuries requiring emergency treatment are treated according to the pelvic fracture management algorithm (Fig. 1). We classifies the patient’s response to initial fluid resuscitation according to Advanced Trauma Life Support (ATLS) [17]. There are three possible patterns of response to the initial fluid bolus: rapid response, transient response, and minimal or no response. Indication for TAE is intrapelvic contrast extravasation or hematoma in a computed tomography scan or a transient responder with hemodynamic instability (HI) associated with pelvic fractures. If needed, TAE is also conducted after pelvic packing or any damage control operation or procedures (Fig. 1).

### Study population

**Fig. 2:**
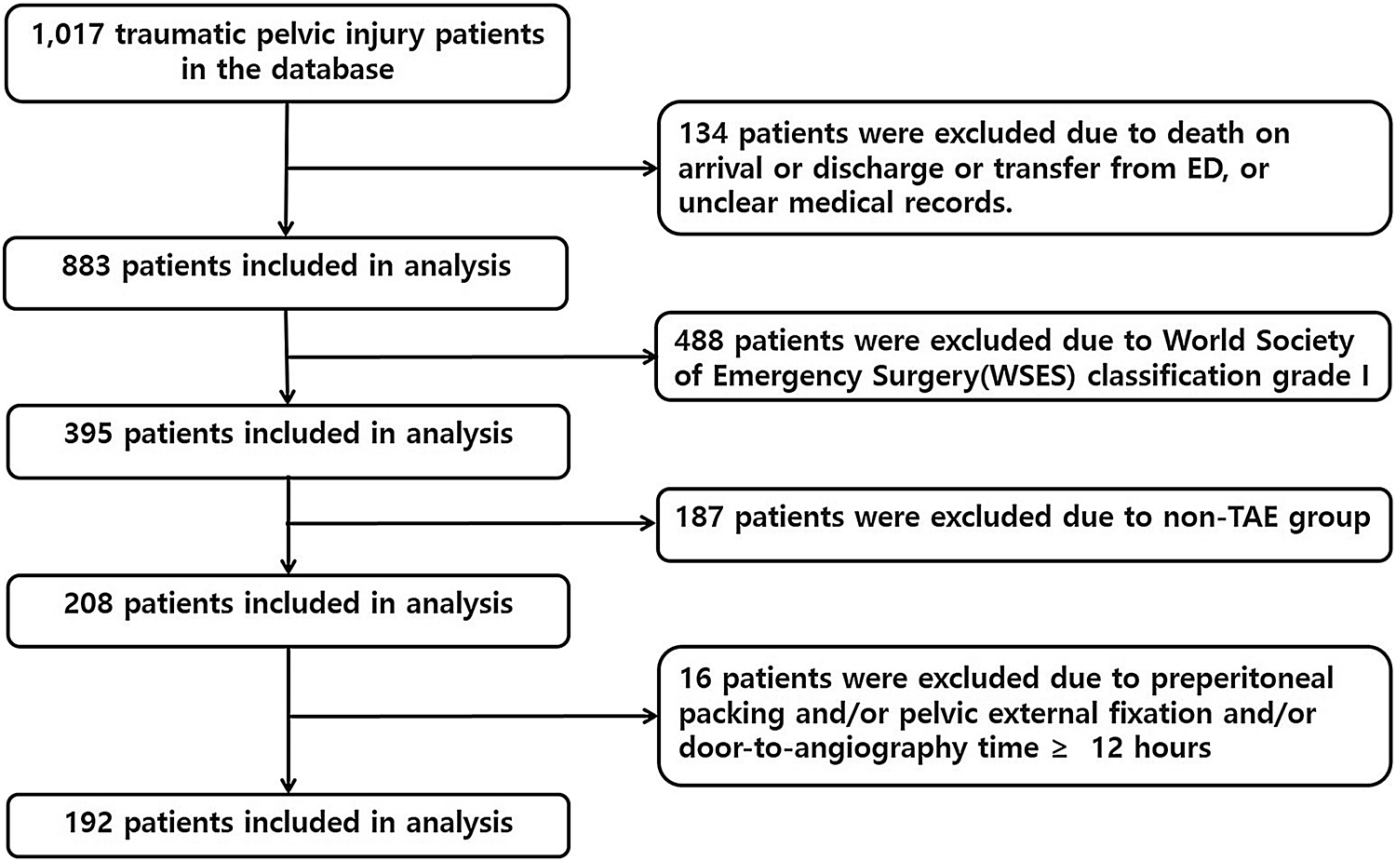
Flowchart of the study. *ED* emergency department, *TAE* transarterial embolization

We retrospectively reviewed data from the medical records and included a total of 1017 patients with pelvic fracture admitted to the trauma resuscitation unit at our Trauma Center between November 1, 2015 and December 31, 2019. Inclusion criteria were adult patients (≥ 18 years), ISS ≥ 16, and patients with an the World Society of Emergency Surgery (WSES) classification of pelvis grade ≥ II (Table E1) [7]. Pelvic injuries almost always accompany injuries to other organ systems. Considering only isolated pelvic injuries would not be realistic; thus, polytraumatic patients with pelvic bone fracture were included in this study. Patients declared dead-on-arrival or discharged or transferred from a trauma resuscitation unit within 24 h or with unclear medical records, patients who did not undergo TAE, patients underwent preperitoneal packing (PPP) and/or pelvic external fixation, and patients underwent angiography more than 12 h after admission were excluded. PPP and/or pelvic fixation were considered as hemostatic intervention, so patients underwent PPP and/or pelvic external fixation were excluded, because they can cause bias of results in this study. In addition, patients underwent angiography more than 12 h after admission were excluded, as they likely had delayed presentation of the indications for TAE or had prolonged periods of time with operative treatment of multiple injuries. The final study population included 192 TAE patients (Fig. 2).

Available data included age, sex, mechanism of injury, vital sign on arrival, transfusion with packed red blood cells (pRBCs) within 4 h and 24 h of arrival, AIS, ISS, Glasgow Coma Scale score (GCS), Revised Trauma Score (RTS), shock index, Trauma and Injury Severity Score (TRISS), massive transfusion within initial 24 h of arrival, hospital length of stay, intensive care unit (ICU) stay, and survival status in the first 24 h, at 28 days, and discharge. Massive transfusion was defined as the replacement by transfusion of 10 units of red blood cells in 24 h.

### Definitions and outcome measures

**Fig. 3:**
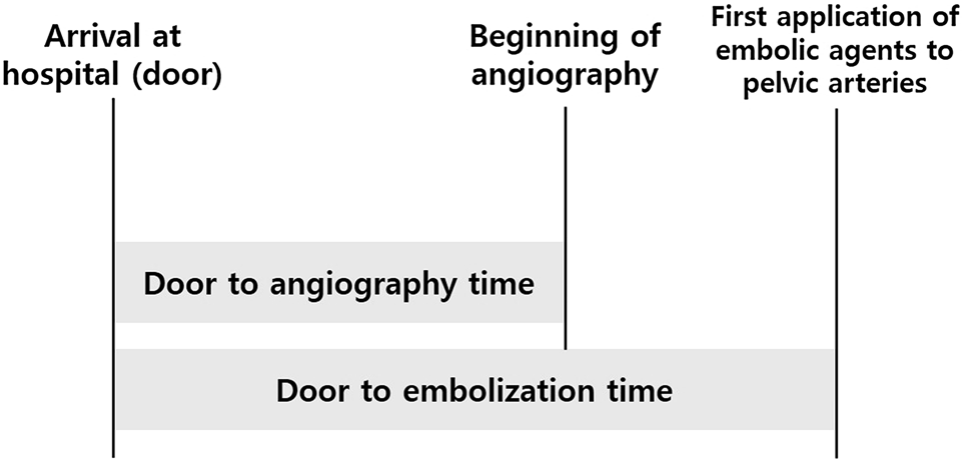
Scheme for timeframe from injury onset to transarterial embolization in trauma patients with severe pelvic fracture

We defined DTE time as the time from the arrival at hospital to the first application of embolic agents such as polyvinyl alcohol, Gelfoam, coils, and so forth to pelvic arteries. We defined door-to-angiography (DTA) time as that from the arrival at the hospital to the beginning of angiography (Fig. 3). Complex pelvic fracture was defined as a pelvic fracture with WSES grade ≥ II in polytrauma patients (Table E1) [7]. The shock index was defined as heart rate (beat/min)/systolic blood pressure (SBP; mmHg). HI was defined as SBP < 90 mmHg and SI ≥ 1.0 on arrival [18, 19]. Daytime was defined as 8:30 a.m. through 5:30 p.m., and the weekend was defined as 5:31 p.m. Friday through 08:29 a.m. Monday.

The primary outcomes were mortality in the first 24 h. Secondary outcomes included overall mortality (in-hospital mortality), pRBC transfusion amounts during the initial 24 h, ICU-free days to day 28, and hospital-free days to day 90. ICU-free days to day 28 were calculated as 28 minus the number of days or part-days in the ICU. All patients who died before the day 28 follow-up were counted as having zero ICU-free days, on the basis that they should be counted as having the worst possible outcome. Hospital-free days to day 90 are a composite of in-hospital death and hospital length of stay, defined as the number of days alive and out of the hospital between the index visit to the trauma resuscitation unit and 90 days later. Patients who died during the index hospitalization and those hospitalized for more than 90 days were classified as having zero hospital-free days. For patients discharged alive before day 90, the number of hospital-free days was calculated as 90 minus the length of stay.

We divided the patients into two groups according to their DTE time (≤ 150 min vs. > 150 min) to assess the effects of that on clinical outcomes. We arbitrarily set the cut-off point (150 min) at the median of the DTE time.

### Statistical analyses

We present continuous variables as median and interquartile ranges and categorical variables as numbers and percentages. We compared categorical variables using the chi-square test when appropriate; otherwise, we used Fisher’s exact test. We compared continuous variables with a Wilcoxon rank-sum test on the basis of the distribution. Multiple binomial logistic regression analyses were performed in a stepwise fashion, evaluating the effects on mortality of DTE time, and injury-to-embolization time. In addition to comparing survival in the first 24 h between DTE time and mortality, Kaplan–Meier plots of survival curves up to 28 days for each group were drawn and their differences were assessed using the log-rank test. We used Cox proportional-hazards model to estimate the hazard ratio of DTE time for mortality at day 28 by adjusting for compounding factors. We performed multiple linear regression analyses to estimate the impact of DTE time on ICU-free days to day 28, hospital-free days to day 90, and 24 h pRBC transfusion requirement. A value of *p* < 0.05 was declared to be statistically significant. The Statistical Package for the Social Sciences (Version 20.0, SPSS, Inc., Chicago, IL, USA) and STATA software (Version 14.2, Stata Corp., College Station, TX, USA) were used to analyze the data.

## Results

### Demographics of patients with severe pelvic fracture undergoing TAE

The median DTA time was 108 min (interquartile range [IQR], 78–134). DTA time was not significantly different between daytime and nighttime (98 min [IQR, 73–134] vs. 112 min [IQR, 86–135], *p* = 0.067) or between weekday and weekend or holiday (101 min [IQR, 77–133] vs. 113 min [IQR, 82–139], *p* = 0.156). The median DTE time was 150 min (IQR, 123–186). The median age was 58 years (IQR, 41–70), and 45.3% were female. The median ISS was 33 (IQR, 25–41) and 32.3% had HI. Most patients had associated severe injuries (AIS ≥ 3), with head and neck (27.1%), thoracic (54.7%), and abdominal (34.9%) injuries occurring most commonly. The median ICU-free days to day 28 and hospital-free days to day 90 were 21 days (IQR, 3–26) and 48 days (IQR, 0–63), respectively. The median 24 h transfusion requirements were five packs (IQR, 2–11) of pRBCs. In addition, the mortality rates in the first 24 h and overall were 3.7% and 15.7%, respectively. The demographics of the patients with complex pelvic fracture undergoing TAE are shown in Table 1. The characteristics of patients died within 24 h of hospital admission is demonstrated in Table E2.

**Table 1:** Characteristics of patients treated with transarterial embolization (*n* = 192)

Characteristics	Variable
Door-to-angiography time, median (IQR), min	106 (78–134)
Door-to-embolization time, median (IQR), min	150 (121–184)
Origin of admission, *n* (%)	
Scene	92 (47.9)
Transfer	100 (52.1)
Time of admission	
Weekday or day, *n* (%)	58 (30.2)
Weekend or night or holiday, *n* (%)	134 (69.8)
Age, median (IQR), years	58 (41–70)
Female, *n* (%)	87 (45.3)
Injury mechanism, *n* (%)	
Car TA	13 (6.8)
Motorcycle TA	19 (9.9)
Pedestrian TA	69 (35.9)
Fall	67 (34.9)
Entrapment	12 (6.2)
Others	12 (6.2)
Physiology at admission	
Systolic blood pressure, median (IQR), mmHg	90 (70–100)
Heart rate, median (IQR), beats/min	94 (80–113)
Shock index, median (IQR)	1.0 (0.8–1.4)
Hemodynamic instability, *n* (%)	62 (32.3)
Lactic acid, median (IQR), mmol/L	3.8 (2.4–6.3)
Base excess, median (IQR)	− 4.0 (− 7.6 to − 0.9)
ISS, median (IQR)	33 (25–41)
GCS, median (IQR)	15 (11–15)
RTS, median (IQR)	7.33 (6.38–7.84)
TRISS score, median (IQR)	0.83 (0.62–0.94)
WSES grade, *n* (%)	
II	25 (13.0)
III	105 (54.7)
IV	62 (32.3)
Head and neck AIS ≥ 3, *n* (%)	52 (27.1)
Chest AIS ≥ 3, *n* (%)	105 (54.7)
Abdomen AIS ≥ 3, *n* (%)	67 (34.9)
Any surgery, *n* (%)	173 (90.1)
Any surgery within 24 h, *n* (%)	67 (34.9)
Pelvis surgery within 24 h, *n* (%)	15 (7.8)
Outcome	
28-day free ICU stay, median (IQR), days	21 (3–26)
90-day free hospital stay, median (IQR), days	48 (0–63)
pRBC transfusion	
≤ 4 h pRBC transfusion, median (IQR), packs	3 (1–6)
4–24 h pRBC transfusion, median (IQR), packs	2 (0–4)
24 h pRBC transfusion, median (IQR), packs	5 (2–11)
MT within 4 h (≥ 10 packs pRBC), *n* (%)	13 (6.8)
MT between 4–24 h (≥ 10 packs pRBC), *n* (%)	19 (9.9)
MT within 24 h (≥ 10 packs pRBC), *n* (%)	52 (27.1)
Mortality within 24 h, *n* (%)	7 (3.7)
Overall mortality, *n* (%)	28 (14.6)
Hemorrhage, *n* (%)^*^	7 (25)
Sepsis or organ failure, *n* (%)^*^	11 (39.3)
Traumatic brain injury, *n* (%)^*^	8 (28.6)
Others, *n* (%)^*^	2 (7.1)

Values are presented as numbers (%) or medians (interquartile range)

*IQR* interquartile range, *TA* traffic accident, *ISS* Injury Severity Score, *GCS* Glasgow Coma Scale, *RTS* Revised Trauma Score, *AIS* Abbreviated Injury Scale, *TRISS* Trauma and Injury Severity Score, *WSES* World Society of Emergency Surgery, *pRBC* packed red blood cells, *ICU* intensive care unit, *MT* massive transfusion

*Attributable percentage of total mortality

### Risk factors for mortality in the first 24 h (Table 2)

**Table 2:** Univariable and multiple logistic regression analyses for mortality in the first 24 h (*n* = 192)

Variable	Crude odds ratio (95% CI)	*p *value	Adjusted odds ratio^*^ (95% CI)	*p *value
Door-to-embolization time, median (IQR), h	1.68 (1.19–2.38)	0.003	2.00 (1.20–3.34)	0.008
Door-to-angiography time, median (IQR), h	1.74 ( 1.22–2.48)	0.002		
Age, median (IQR), years	1.00 (0.94–1.07)	0.908		
Female, *n* (%)	0.60 (0.05–6.71)	0.678		
Physiology at admission				
Systolic blood pressure, median (IQR), mmHg	0.97 (0.95–1.00)	0.120		
Heart rate, median (IQR), beats/min	0.94 (0.91–0.98)	0.003		
Hemodynamic instability, *n* (%)	0.85 (0.07–9.54)	0.895		
Lactic acid, median (IQR), mmol/L	1.10 (0.96–1.27)	0.179		
Base excess, median (IQR)	0.88 (0.76–1.01)	0.075		
ISS, median (IQR)	1.12 (0.99–1.25)	0.062		
GCS, median (IQR)	0.73 (0.56–0.97)	0.027	0.63 (0.40–0.98)	0.040
RTS, median (IQR)	0.99 (0.44–2.22)	0.987		
TRISS, median (IQR)	0.003 (0.000–0.466)	0.023		
Head and neck AIS ≥ 3, *n* (%)	1.35 (0.12–15.24)	0.807		
Chest AIS ≥ 3, *n* (%)	1.67 (0.15–18.73)	0.678		
Abdomen AIS ≥ 3, *n* (%)	3.81 (034–42.87)	0.278		
pRBC transfusion within 24 h, median (IQR), packs	1.05 (1.01–1.09)	0.013		

Values are presented as numbers (%) or medians (interquartile range)

*CI* confidence interval, *IQR* interquartile range, *ISS* Injury Severity Score, *GCS* Glasgow Coma Scale, *RTS* Revised Trauma Score, *TRISS* Trauma and Injury Severity Score, *AIS* Abbreviated Injury Scale, *pRBC* packed red blood cells

*Adjusted odds ratio for age, gender, systolic blood pressure, ISS, GCS, and pRBC transfusion in the initial 24 h

In univariate analyses, factors associated with mortality in the first 24 h of patients with complex pelvic fracture were DTE time, DTA time, heart rates upon admission, GCS, TRISS, and pRBC transfusion amounts in the initial 24 h. Considering clinical priority and statistical significance, we finally selected 6 confounding factors (age, gender, SBP upon arrival, ISS, GCS, and pRBC transfusion amounts during the initial 24 h) in multiple binomial logistic analysis. After adjusting for the six variables, DTE time was an independent risk factor for mortality in the first 24 h. An increase of 1 h in DTE time resulted in a 2.00–fold increase in mortality in the first 24 h.

Cox proportional hazards regression analyses were performed to evaluate the independent risk factors for mortality at 28 days. After adjusting for age, SBP upon arrival, ISS, GCS, TRISS, and pRBC transfusion amounts during the initial 24 h, the adjusted hazard ratio of DTE time was 1.24. This means that an increase of 1 h in DTE time resulted in a 1.24-fold increase in mortality at 28 days.

### Secondary outcomes of patients according to DTE time

**Fig. 4:**
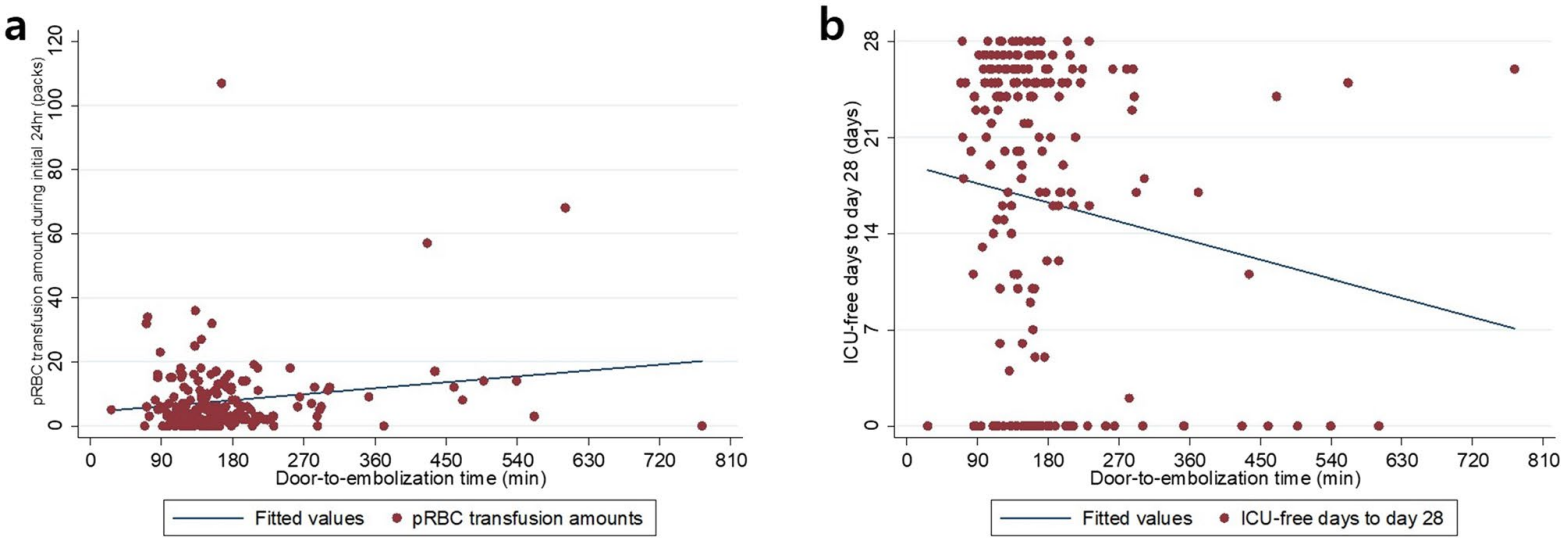
Relationships between **a** door-to-embolization time and the requirement for packed red blood cells transfusion requirement in the initial 24 h, and **b** door-to-embolization time and ICU-free days to day 28. *pRBC* packed red blood cells

Multiple linear regression analyses were performed to evaluate the effects of DTE time on pRBC transfusion requirement in the initial 24 h, ICU-free days to day 28, and hospital-free days to day 90. DTE time was an independent indicator of 24 h pRBC transfusion requirement and ICU-free days to day 28 (*p* = 0.012 and 0.025, respectively; Table 3). Figure 4 shows the positive relationship between DTE time and pRBC transfusion amounts in the initial 24 h and the negative relationship between DTE time and ICU-free days to day 28. However, no significant difference in hospital-free days to day 90 was found. Similarly, overall mortality was not significantly different in multiple logistic regression analyses (adjusted OR = 1.28, 95% CI = 0.97–1.68, *p* = 0.082).

**Table 3:** Secondary outcomes according to door-to-embolization time (*n* = 192)

	Unstandardized coefficient^†^	Standard error	Standardized coefficients beta^†^	*p *value	Crude OR (95% CI)	*p *value	Adjusted OR (95% CI) ^‡^	*p *value
pRBC transfusion requirement in the initial 24 h^*^	1.21	0.480	0.172	0.012				
ICU-free days to day 28^*^	− 0.921	0.407	− 0.138	0.025				
Hospital-free days to day 90^*^	− 1.519	1.142	− 0.087	0.185				
Overall mortality^*^					1.29 (1.06–1.58)	0.012	1.28 (0.97–1.68	0.082

*OR* odds ratio, *CI* confidence interval, *pRBC* packed red blood cells, *ISS* Injury Severity Score, *AIS* Abbreviated Injury Scale, *GCS* Glasgow Coma Scale, *RTS* revised trauma scale

*Door-to-embolization time per 1 h increase. †Adjusted coefficient for ISS, age, hemodynamic instability, and AIS for pelvic ring fracture. ‡adjusted odds ratio for base excess, ISS, GCS, TRISS and pRBC transfusion in the initial 24 h

### Subgroup analyses: clinical outcomes according to DTE time (≤ 150 min vs. > 150 min)

**Fig. 5:**
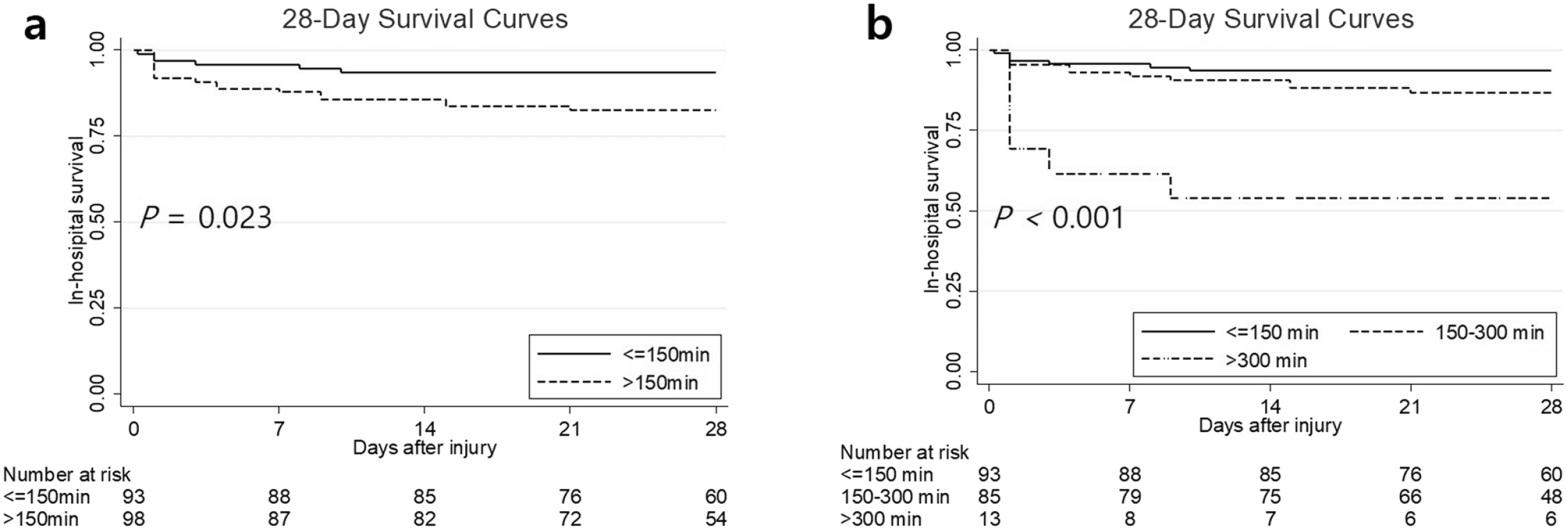
Kaplan–Meier 28-day mortality curves of patients treated with transarterial embolization according to door-to-embolization time

We divided the patients into two groups according to their DTE time to assess the effects of this factor on clinical outcomes. We set the cut-off point as 150 min, which was the median DTE time. Figure 5 shows the Kaplan–Meier 28-day mortality curves of patients undergoing TAE according to DTE time. The incidence of 28-day mortality was significantly lower in patients with DTE time ≤ 150 min than in patients with DTE time > 150 min (*p* = 0.023) and a similar result remained even after we divided patients into three groups according to their DTE time (≤ 150 vs. 150–300 vs. > 300 min; *p* < 0.001; Fig. 5).

## Discussion

The main findings of this study are that a shorter DTE time was associated with a lower risk for mortality in the first 24 h. In addition, DTE time was an independent predictor of requirement for pRBC transfusion in the initial 24 h and ICU-free days to day 28. However, no benefits of reducing DTE time were apparent in terms of overall mortality or hospital-free days to day 90.

### Comparison with other studies

The cause of early mortality was often haemorrhage, whereas the cause of late mortality was sepsis/organ failure or traumatic brain injury. Thus, we did not choose survival-to-discharge but instead survival in the first 24 h as a primary outcome to distinguish deaths due to bleeding from others. Early angiography with embolization, when needed, has been shown to improve patient survival [6, 20, 21], and several studies support this hypothesis [10–13, 22]. On the other hand, some have reported that there are no significant differences in DTE time between nonsurviving and surviving patients [14, 15]. Table 4 shows a summary of reported series about the impact of DTE time on the mortality of patients with pelvic fracture undergoing TAE [10–15, 22]. In our study, shortening DTE time was associated with better survival in the first 24 h as well as other clinical outcomes in patients with complex pelvic fracture undergoing TAE. Early access to angiography is associated with reduced mortality. Longer DTE time is associated with worse outcomes [10–15, 22]. Therefore, efforts to minimize DTE time are recommended to improve clinical outcomes in patients with pelvic fracture treated with TAE [6, 14]. Recently, Ito et al. [23] reported that a hybrid emergency room system improved the timeliness of TAE for pelvic fracture.

**Table 4:** Summary of reported series about the impact of door-to-embolization time on the mortality of patients with pelvic fracture undergoing transarterial embolization

Study citation (year)	No. of TAE cases	Outcome variable	Time (min)	Impact on mortality
Agolini et al. [11]	15	Time from arrival to angiography suite	190 min (IQR, 50–1440)	Patients who were in the angiography suite within 3 h of arrival had a significantly greater survival rate (14 vs. 75%)
Balogh et al. [12]	31	DTA time	< 90 min after admission	Institutional protocol improving time to angiography to less than 90 min decreased mortality from 35 to 7% (*p* < 0.05)
Schwartz et al. [22]	88	Time from admission to angiography suite	Day: 193 min (IQR, 137–275), after-hours: 301 min (IQR, 211–389)	Delays to angiography in after-hours admission were associated with higher mortality (32 vs. 21%, *p* = 0.328)
Tanizaki et al. [13]	68	Time from arrival to angiography suite	Average of 76 min (30–145)	Patients who were embolized within 60 min of arrival had a significantly lower mortality rate (16 vs. 64%, *p* = 0.04)
Tesoriero et al. [15]	212	DTA time	280 min (IQR, 201–367)	Time to angiography was not a significant contributor to mortality after adjusting for injury severity
Marsushuma et al. [10]	181	DTE time	(Not applicable)	A longer time to TAE was significantly associated with increased in-hospital mortality (OR = 1.79 for each hour, 95% CI = 1.12–2.91, *p* = 0.018)
Chou et al .[14]	84	DTE time	62.0 ± 33.4 min	There were no significant differences in the time to TAE between nonsurviving and surviving patients (76.9 ± 47.9 vs. 59.0 ± 29.3 min, p = 0.068)
This study (2020)	204	DTE time	150 min (IQR, 123–186)	An increase in 1 h in door-to-embolization time resulted in a 2.00-fold increase in mortality in the first 24 h (*p* = 0.008)

*TAE* transarterial embolization, *IQR* interquartile range; *DTA* door to angiography, *DTE* door to embolization, *NA* not applicable, *OR* odds ratio, *CI* confidence interval

In this study, we further analyzed how DTE time affected the requirement for pRBC transfusion, ICU-free days to day 28, and hospital-free days to day 90. We found that there were trends in which increasing DTE time resulted in both a higher requirement for 24 h pRBC transfusion and fewer ICU-free days to day 28; the data in Table 3 and Fig. 4 show these two relationships. These results support other results that shortening DTE reduces the 24 h transfusion requirement and ICU length of stay [12, 14].

### Implications of study

There are three main differences between our study and other studies that have suggested that shortening DTE time might be associated with better clinical outcomes. First, we clearly defined DTE and DTA times; we believe that these should be clearly distinguished because of their different effects on the clinical outcomes. However, many studies did not have a clearly defined DTE or DTA, and the two occasionally appeared to be used interchangeably [10–15, 22]. By contrast, we clearly defined DTE time as that from arrival at hospital to the first application of embolic agents to pelvic arteries and DTA time as that from arrival at hospital to the beginning of angiography. The current study showed that there was a continuous association between shortening DTE time and reduced risk for mortality. Second, our study suggested that shortening DTE time could reduce patients’ mortality rate as well as other outcomes, such as blood transfusion requirement and ICU length of stay. On the other hands, other study showed only reduced mortality or reduced transfusion require or decreased hospital stay [10–15, 22]. We thought major novelty of this study was the impact of shorter DTE time on various clinical outcomes in patients with complex pelvic fracture who underwent TAE. Third, the median DTE time in this study was shorter than that in other studies. Despite the suggestion that short DTE time improves mortality, some reported series document considerable delays. Gannslen et al. [24] reported that the average DTE time was 10.7 h. Tesoriero et al. [15] reported that the median time to embolization was 344 min (4.7 h). Evers et al. [25] reported that the mean time from admission to angiography was more than 4 h. By contrast, the median DTA time in our study was 107 min (1.8 h) and the median DTE time was 150 min (2.5 h). In addition, the DTA time was not significantly different between daytime and nighttime (98 min [IQR, 73–134] vs. 112 min [IQR, 86–135], *p* = 0.067) or between weekday and weekend or holiday (101 min [IQR, 77–133] vs. 113 min [IQR, 83–139], *p* = 0.156). Our three interventional radiologists and the equipment required for TAE are available around the clock [16]. Thus, we believe that DTA time and DTE time can be shortened and our results indicate that patients with a short DTE time (≤ 150 min) achieved better clinical outcomes.

### Limitations of study

There were two limitations of this study. First, it was confined to patients at a single center and our study population may not represent general patients with pelvic fracture. For example, our study population consisted of polytraumatic patients who did not undergo PPP and/or pelvic fixation and was limited to complex pelvic fracture (WSES grade ≥ II) [7], which may limit the generalizability of our findings. Second, because this was a non-randomized, retrospective analysis, the results are not conclusive. Additional, prospective, randomized, controlled trials with a larger sample size are necessary to verify our findings.

## Conclusion

Shorter DTE time was associated with better clinical outcomes in patients with complex pelvic fracture who underwent TAE. These findings suggest that shortening DTE time could reduce patients’ mortality rate as well as other outcomes, such as blood transfusion requirement and ICU length of stay. Thus, DTE time is an important factor to consider when treating patients with suspected pelvic hemorrhage. Efforts to minimize DTE time are recommended to improve the clinical outcomes in patients with pelvic fracture treated with TAE.

## Supplementary Information

Below is the link to the electronic supplementary material.

Supplementary file1 (DOCX 14 KB)

Supplementary file2 (DOCX 16 KB)
