# Supplementary material for: Relationship between door-to-embolization time and clinical outcomes after transarterial embolization in trauma patients with complex pelvic fracture
Source: Eur J Trauma Emerg Surg. 2021 Feb 1;48(3):1929–38. doi: 10.1007/s00068-021-01601-7 (PMC9192384; doi:10.1007/s00068-021-01601-7)
Supplement: Supplementary file 1 — Supplementary file1 (DOCX 14 KB) [file 68_2021_1601_MOESM1_ESM.docx]

Table E1. World Society of Emergency Surgery (WSES) pelvic injuries classification [7]

|  | WSES grade | Young-Burgees classification | Haemodynamic | Mechanic | First-line Treatment |
| --- | --- | --- | --- | --- | --- |
| Minor | WSES grade I | APC I, LC I | Stable | Stabe | NOM |
| Moderate | WSES grade II | APC II/III, LC II/III | Stable | Unstable | Pelvic binder in the field ± Angioembolization (if blush at CT-scan)  OM – Anterior External fixation |
|  | WSES grade III | VS, CM | Stable | Unstable | Pelvic Binder in the field  ± Angioembolization (if blush at CT-scan)  OM - C-Clamp * |
| Severe | WSES grade IV | Any | Unstable | Any | Pelvic Binder in the field  Preperitoneal Pelvic Packing  ± Mechanical fixation (see below)  ± REBOA  ± Angioembolization |

*: patients hemodynamically stable and mechanically unstable with no other lesions requiring treatment and with a negative CT-scan, can proceed directly to definitive mechanical stabilization.

LC, Lateral Compression; APC, Antero-posterior Compression; VS, Vertical Shear; CM, Combined Mechanism; NOM, Non-Operative Management; OM, Operative Management; REBOA, Resuscitative Endo-Aortic Balloon
