# Supplementary material for: Relationship between door-to-embolization time and clinical outcomes after transarterial embolization in trauma patients with complex pelvic fracture
Source: Eur J Trauma Emerg Surg. 2021 Feb 1;48(3):1929–38. doi: 10.1007/s00068-021-01601-7 (PMC9192384; doi:10.1007/s00068-021-01601-7)
Supplement: Supplementary file 2 — Supplementary file2 (DOCX 16 KB) [file 68_2021_1601_MOESM2_ESM.docx]

Table E2. Characteristics of patients with the 24-hour mortality (n = 7)

| Characteristics | Variable |
| --- | --- |
| Door-to-angiography time, median(IQR), min | 124 (71 – 421) |
| Door-to-embolization time, median (IQR), min | 155 (94 – 460) |
| Age, median (IQR), years | 72 (53 – 80) |
| Female, n (%) | 2 (28.6) |
| Injury Mechanism, n (%) |  |
| Car TA | 0 |
| Motorcycle TA | 2 (28.6) |
| Pedestrian TA | 4 (57.1) |
| Fall | 0 |
| Entrapment | 1 (14.3) |
| Others | 0 |
| Physiology at admission |  |
| Systolic blood pressure, median (IQR), mmHg | 60 (40 – 90) |
| Heart rate, median (IQR), mmHg | 106 (66 – 142) |
| Shock index, median (IQR) | 2.0 (1.2 – 2.6) |
| Hemodynamic instability, n (%) | 6 (85.7) |
| Lactic acid, median (IQR) | 9.0 (8.5 – 13.1) |
| Base excess, median (IQR) | -10.7 (-18.5 – -7.4) |
| ISS, median (IQR) | 38 (34 – 45) |
| GCS, median (IQR) | 7 (5 – 12) |
| RTS, median (IQR) | 6.37 (3.77 – 6.90) |
| TRISS score, median (IQR) | 0.46 (0.31 – 0.53) |
| WSES grade, n (%) |  |
| II | 0 |
| III | 4 (57.1) |
| IV | 3 (42.9) |
| pRBC transfusion |  |
| ≤4 h pRBC transfusion, median (IQR), packs | 17 (9 – 28) |
| 4–24 h pRBC transfusion, median (IQR), packs | 4 (2 – 40) |
| 24 h pRBC transfusion, median (IQR), packs | 18 (12 – 68) |
| MT within 4 h (≥10 packs pRBC), n (%) | 5 (71.4) |
| MT between 4–24 h (≥10 packs pRBC), n (%) | 2 (28.6) |
| MT within 24 h (≥10 packs pRBC), n (%) | 6 (85.7) |

Values are presented as numbers (%) or medians (interquartile range).

IQR, Interquartile range; TA, traffic accident; ISS, Injury Severity Score; GCS, Glasgow Coma Scale; RTS, Revised Trauma Score; TRISS, Trauma and Injury Severity Score; WSES, World Society of Emergency Surgery; pRBC, Packed red blood cells; MT, Massive transfusion
